# Attenuation Aβ_1-42_-induced neurotoxicity in neuronal cell by 660nm and 810nm LED light irradiation

**DOI:** 10.1371/journal.pone.0283976

**Published:** 2023-07-21

**Authors:** Siriluk Thammasart, Poommaree Namchaiw, Kwanchanok Pasuwat, Khaow Tonsomboon, Anak Khantachawana

**Affiliations:** 1 Biological Engineering Program, Faculty of Engineering, King Mongkut’s University of Technology Thonburi (KMUTT), Thung Kru, Bangkok, Thailand; 2 Neuroscience Center for Research and Innovation, Learning Institute, King Mongkut’s University of Technology Thonburi (KMUTT), Thung Kru, Bangkok, Thailand; 3 Department of Chemical Engineering, Faculty of Engineering, King Mongkut’s University of Technology Thonburi (KMUTT), Thung Kru, Bangkok, Thailand; 4 National Center for Genetic Engineering and Biotechnology (BIOTEC), National Science and Technology Development Agency (NSTDA), Pathum Thani, Thailand; 5 Department of Mechanical Engineering, Faculty of Engineering, King Mongkut’s University of Technology Thonburi (KMUTT), Thung Kru, Bangkok, Thailand; Institut d’Investigacions Biomediques de Barcelona, SPAIN

## Abstract

Oligomeric amyloid-β 1–42 (Aβ_1–42_) has a close correlation with neurodegenerative disorder especially Alzheimer’s disease (AD). It induces oxidative stress and mitochondrial damage in neurons. Therefore, it is used to generate AD-like *in vitro* model for studying neurotoxicity and neuroprotection against amyloid-β. A low-level light therapy (LLLT) is a non-invasive method that has been used to treat several neurodegenerative disorders. In this study, the red wavelength (660nm) and near infrared wavelength (810nm) at energy densities of 1, 3, and 5 J/cm^2^ were used to modulate biochemical processes in the neural cells. The exposure of Aβ_1–42_ resulted in cell death, increased intracellular reactive oxygen species (ROS), and retracted neurite outgrowth. We showed that both of LLLT wavelengths could protect neurons form Aβ_1-42_-induced neurotoxicity in a biphasic manner. The treatment of LLLT at 3 J/cm^2^ potentially alleviated cell death and recovered neurite outgrowth. In addition, the treatment of LLLT following Aβ_1–42_ exposure could attenuate the intracellular ROS generation and Ca^2+^ influx. Interestingly, both wavelengths could induce minimal level of ROS generation. However, they did not affect cell viability. In addition, LLLT also stimulated Ca^2+^ influx, but not altered mitochondrial membrane potential. This finding indicated LLLT may protect neurons through the stimulation of secondary signaling messengers such as ROS and Ca^2+^. The increase of these secondary messengers was in a functional level and did not harmful to the cells. These results suggested the use of LLLT as a tool to modulate the neuronal toxicity following Aβ_1–42_ accumulation in AD’s brain.

## Introduction

Alzheimer’s disease (AD) is the most common neurodegenerative disorder in elderly. The etiology of AD is remained unknown but believed to be correlated with the abnormal accumulation of amyloid-β (Aβ) in the brain. The neurotoxicity of amyloid-β was correlated with its structure. Previous studies revealed that the oligomeric Aβ had high affinity binding to synaptic contact and cellular membrane of human cortical neurons. This binding led to Mitochondria-mediated apoptosis [1, 2]. A study done by Morkuniene et al. revealed that oligomeric Aβ induce glutamate toxicity in neuron. The increase of extracellular glutamate, in turn, led to the induction of calcium influx and ultimately cell death [3]. The molecular mechanism revealed that oligomeric Aβ enhance the calcium release from mitochondria through the mitochondrial permeability transition pore (mPTP) and thus initiate apoptosis pathway [4].

Ca^2+^ and Aβ intersect at several functional levels and temporal stages of AD. The disruption of Ca^2+^ homeostasis induced by oligomeric Aβ is implicated in diverse disease processes and has become a major focus of study in multifactorial neurodegenerative diseases [5]. Oligomeric Aβ exacerbates the increased resting cytosolic Ca^2+^ level observed in aging neurons [6]. The resulting excessive increase in Ca^2+^ enhances the production of reactive oxygen species (ROS), decreases mitochondrial potential, impairs function of membrane ATPases, and resulting in neuronal dysfunction, such as neurite retraction, synaptic loss, and ultimately neuronal death [7, 8].

Aβ toxicity in human SH-SY5Y cells has been studied extensively in many previous studies. In this work, SH-SY5Y neuroblastoma cells were differentiated using retinoic acid (RA) and brain-derived neurotrophic factor (BDNF), which is a simple model suggested for neuronal screening [9, 10], to investigate the effects of Aβ_1–42_ oligomers. Consistent with previous studies, our results support the evidence that Aβ_1–42_ oligomers cause cell death by inducing neurite outgrowth retraction and increasing ROS production, which is related to the decrease in mitochondrial membrane potential (MMP) and the overload of intracellular calcium.

Low-level light therapy (LLLT) involves the application of low-intensity light to living cells for therapeutic purposes. It is introduced as a non-invasive and drug-free therapeutic approach that stimulates cells to generate more energy, promote reduction of inflammation and undergo self-repair, regeneration, and restoration of various organism functions [11]. In recent years, LLLT using the irradiation in the wavelength range of 600–900 nm has been increasingly introduced as a neuroprotective approach to treat neurodegenerative disease. This therapy has been shown to alleviate dopaminergic neuron death in animal models of Parkinson disease (PD), and to reduce both Aβ and tau protein levels, both *in vitro* and *in vivo*, for AD [12]. Red and near-infrared (NIR) are popular for LLLT due to their efficient absorption by biological tissues and the beneficial effects of intracellular signal modulation or treatment. The effective application of red and NIR light relies on their ability to penetrate deeply into the body and exert their effects on photoreceptors within the cell, which is facilitated by the reduced light scattering properties [13, 14].

The mechanism of LLLT in cellular processes and intracellular pathways associated with cytochrome c oxidase (CCO), a mitochondrial protein complex that absorbs light in the range of 600–900 nm. LLLT produces a CCO redox state change, which activates a transient alteration in cellular stress response systems in the electron transport chain of mitochondria [15]. This increase in proton pumps and generate MMP, an essential component in the process of energy storage during oxidative phosphorylation. Moreover, LLLT’s ability to increase MMP induces a moderate burst in ROS resulting in the activation of neuroprotective mechanisms, an increase in cell viability, the formation of intracellular signaling, changes in neural cell metabolism, and prompting of apoptosis [16].

The generation of ROS, which activates Ca^2+^ release from endoplasmic reticulum, is considered a possible light-dependent signaling pathway [17]. Red and NIR light have been reported to affect mitochondrial activity by enhancing membrane potential and Ca^2+^ uptake with a biphasic pattern [18, 19]. The activation of calcium influx occurs indirectly due to ROS and MMP production or because of direct stimulation of Ca^2+^ transporters [20, 21]. However, the therapeutic effect of red and NIR LLLT on Aβ_1-42_-induced toxicity in neuron has not been clearly established. This study therefore focuses on the effects of LLLT on Aβ_1-42_-induced toxicity in neuronal cell and other related photoinduced pathways that will elucidate how to apply LLLT effectively for the treatment of AD and other neurodegeneration.

In this study, we examined the impact of LLLT on Aβ_1-42_-induced toxicity in RA/BDNF-differentiated SH-SY5Y cells. Our investigation focused on the possible signaling pathways that contribute to the recovery of neurite retraction and viability. We investigated the effect of 660 and 810 nm light-emitting diodes (LED) at the varying intensities of 1, 3, and 5 J/cm^2^. Our findings indicate that LLLT significantly attenuated Aβ_1-42_-induced neurotoxicity and cell death by modulating mitochondrial activity and promoting neurite outgrowth. Remarkably, in contrast to Aβ_1–42_ toxicity, both 660 and 810 nm light provoked an elevation of ROS and intracellular Ca^2+^ without exerting any toxic effects on the cell nor inducing cell death. It is worth noting that both wavelengths also modulated the mitochondrial depolarization.

## Materials and methods

### Chemicals and reagents

Cell culture associated reagents were purchased from Gibco®/ Invitrogen: Dulbecco’s modified Eagle’s Medium, Ham’s F-12 nutrient mix, 0.25% Trypsin-EDTA solution, antibiotic/antimycotic, Fetal Bovine serums, Trypan Blue stain. MTT cell viability reagent was purchased from GIBCO (Invitrogen Corporation, Grand Island, NY, USA). Human beta amyloid 1–42 (Aβ_1–42_) PTD recombinant protein, Brain derived neurotrophic factor (BDNF), retinoic acid (RA), poly-L-lysine hydrobromide, collagen type I solution, 1,1,1,3,3,3-hexafluoro-2-propanol (HFIP), 4-(2-hydroxyethyl)-1-piperazineethanesulfonic acid (HEPES), 2’,7’-dichlorodihydrofluorescein diacetate (DCFH-DA), JC-1 dye, and Fluo-3/AM were purchased from Sigma-Aldrich.

### SH-SY5Y cell culture

SH-SY5Y neuroblastoma cells were maintained at 37°c in a humidified atmosphere of 5% CO_2_. Cells were grown in a mixture medium of 1:1 Dulbecco Modified Eagle Medium (DMEM) and Ham’s F12 supplemented with 10% fetal bovine serum (FBS) and 1% antibiotic/antimycotic. Culture medium was aspirated every 3 days and cells were passaged once they reached 80% confluence using 0.25% Trypsin-EDTA solution.

### SH-SY5Y differentiation

SH-SY5Y cells were differentiated following the differentiation protocol developed by Forster, J. I., et al. [10]. Briefly, the neuronal differentiation was carried out in two steps using sequential chemical induction of RA in full medium for 5 days, followed by BDNF in serum-free medium for 2 days. RA/BDNF differentiated SH-SY5Y cells will be referred as neuron-like cells in this work. After cell differentiation, the differentiated neurons were incubated with Aβ_1–42_ oligomers for 1 day, and subsequently irradiated with LLLT for 7 days. The media containing Aβ_1–42_ were changed every two days.

### Preparation of amyloid-β 1–42 oligomer

Aβ_1–42_ oligomer was prepared according to the protocol outlined by Stine et al. with slight modification [22]. First, a solution of 1 mg/mL Aβ_1–42_ peptide in HFIP was prepared and incubated at room temperature (RT) for 1h with occasional vortex at moderate speed, followed with a 10 min-sonication in a water bath sonicator. The Aβ-HFIP solution was then dried with nitrogen gas to obtain a clear thin peptide film at the bottom of the tubes. Dried peptide films were stored at -20°C until use. Prior to each experiment, the peptide films were thawed to RT, resuspended in 100% anhydrous Dimethyl Sulfoxide (Sigma-Aldrich, MO, USA) to a final concentration of 5mM, vortexed thoroughly, and sonicated for 10 min. The Aβ_1–42_ solution was further diluted in ice-cooled DMEM Phenol red-free medium to acquire a final concentration of 10 μM.

### Low-level light irradiation

**Fig 1:**
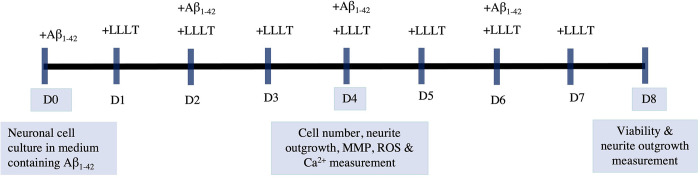
Treatment flow chart. Neuronal cells were incubated with Aβ_1–42_ on day 0, and the media containing Aβ_1–42_ was changed every two days. The LLLT treatment interval was varied, with cells receiving treatment once a day. After treatment with LLLT for 3 or 7 days, cells were assayed at each indicated time point.

An AlGaInP light-emitting diodes (LEDs) at 660 nm with 250 mW output power and AlGaAs LED at 810 nm with 340 mW output power. The LEDs were used by focusing the beam from the LEDs planar array on the top of the culture plate. This study uses MATLAB simulation to design LEDs planar array with a sufficient intensity distribution of light. The LEDs array was initially performed by setting the system to be considered and Cartesian coordinates, see S1–S3 Figs. 660 nm LED and 810 nm LED were placed on the irradiating plane with an output of a constant power density at 5 mW/cm^2^. The differentiated neurons were irradiated with a power density of 5 mW/cm^2^ for different time periods to achieve energy densities of 1, 3, 5 J/cm^2^. This experiment was designed to realize the effect of light over an irradiation time of 7 days (pictorially described in Fig 1).

### Viability assay

The trypan blue dye exclusion test was used to determine the number of viable cells present in a cell suspension. A viable cell will have a clear cytoplasm whereas a nonviable cell will have a blue cytoplasm. To measure cell viability, the 0.4% w/v of trypan blue dye was added to the cell suspension and thoroughly mix by pipetting. The mixture cells were immediately counted by using a hemocytometer. The percentage of viable cells was calculated by dividing the number of viable cells with the number of total cells and then multiplying with 100.

### Measurement of intracellular reactive oxygen species (ROS)

To quantify oxidative stress by measuring total reactive oxygen species (ROS) using 2’,7’-dichlorodihydrofluorescein diacetate (DCFH-DA) staining modified from the instruction of a previous study [23]. After LLLT, all experimental groups were incubated with 10 μM DCFH-DA dissolved in serum free DMEM at 37°C for 30 min. The fluorescence was then taken at 488 nm excitation and 525 nm emission with a fluorescence microscope (Cytell^TM^).

### Measurement of mitochondrial membrane potential (MMP)

Mitochondrial membrane potential was measured using the cationic JC-1 dye as a sensitive fluorescent probe according to the manufacturer’s protocol. In healthy cells with a normal MMP, the JC-1 dye can enter into the mitochondria and accumulate in the energized and negatively charged mitochondria, spontaneously forming red fluorescent J-aggregates. By contrast, in unhealthy or apoptotic cells, the inside of mitochondria is less negative due to increased membrane permeability and consequent loss of electrochemical potential. Thus, JC-1 enters the mitochondria to a lesser extent [24].

After LLLT, cells were incubated with 500 μl JC-1 staining solution at 37°C for 30 min. Following the incubation, the cells were washed once with the warm phosphate-buffer saline (PBS). Finally, the PBS was added into the cultures and then the fluorescence was observed with a fluorescence microscope (Cytell^TM^) using a dual-bandpass filter. Red fluorescence appears under an emission at 590 nm, while green fluorescent appears under an emission at 530 nm.

### Measurement of intracellular calcium

Intracellular Ca^2+^ was determine using the Fluo-3/AM dye, as an indicator of intracellular Ca^2+^ in living cells. After LLLT, the cells were then trypsinized, washed 3 times with PBS, and resuspended in PBS. Subsequently, cell suspensions (100 ml) for Ca^2+^ analysis were loaded with 5 mM Fluo-3/AM for 30 min at 37°C in the dark, washed once with PBS to remove the excessive Fluo-3/AM. PBS, replacing Fluo-3/AM, served as a negative control. Finally, the cells for each example were resuspended in 1 ml PBS, followed by adding the suspension into a 96-well plate (150 ml/well). Fluorescent intensity at 535 nm was recorded using an excitation wavelength at 488 nm with a microplate reader (Infinite® M200, TECAN).

### Software preparation and image analysis

Analysis of neurite outgrowth was performed with ImageJ software according to the protocol outlined by Boulan, Benoit, et al. with some modification [25, 26]. First, all images were prepared by optimizing *Phase/Contrast* and removing the image background. Next, neurite outgrowth was measured by tracing all neurites with NeuronJ toolbar.

### Statistical analysis

All graphs represent mean ± standard error values calculated from data obtained from at least three independent experiments, each of which was performed in triplicate. R program was used to perform one-way analysis of variance (ANOVA) and t-test. Serial or multiple comparisons were conducted using analysis of variance and post-hoc testing, or the Kruskal-Wallis test, for parametrically and nonparametrically distributed values, respectively. A P-value < 0.05 was considered to reflect statistical significance.

## Results

### The exposure of oligomeric Aβ_1–42_ induced neurotoxicity in differentiated neurons

**Fig 2:**
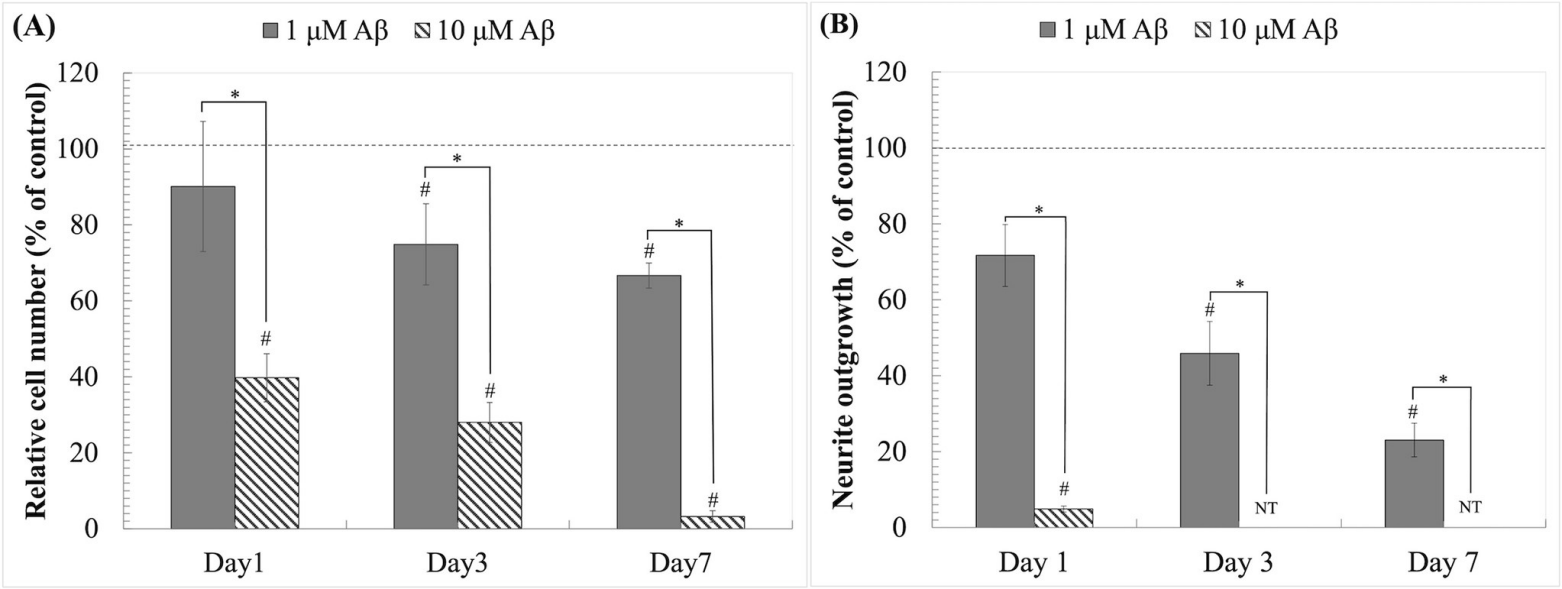
Aβ_1–42_ induces cell death and neurite retraction. (A) Relative cell number (% of control). (B) Neurite outgrowth (% of control). The values are means ± SD (n = 4); *P<0.05 between the determined groups. #P<0.05 vs control. T-test was used for statistical analysis. NT stand for not determine.

**Fig 3:**
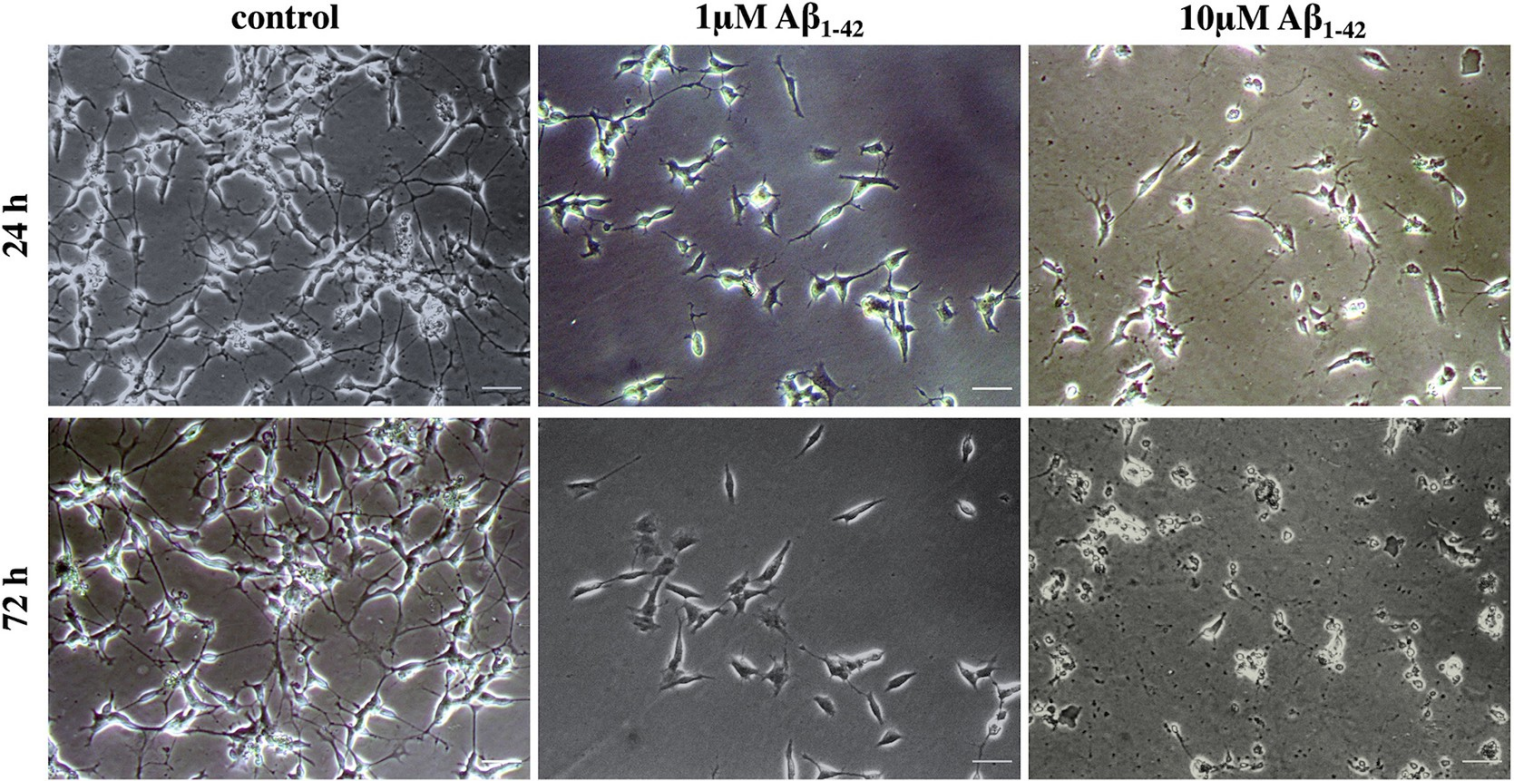
Aβ_1–42_ induces pathological changes in neurite morphology after 24h and 72h. Scale bars represent 50μm.

In this study, neuroblastoma SH-SY5Y cell line was utilized the neurotoxicity against oligomeric Aβ_1–42_. The differentiated neurons were treated with Aβ_1–42_ at concentration of 1 and 10μM for 7 days. The morphological study, cell number and neurite outgrowth were observed at day 1, 3, and 7 to determine Aβ_1-42_-induced neurotoxicity. The results showed that Aβ_1–42_ treatment decreased cell number and neurite outgrowth in a dose- and time-dependent manner (Fig 2). The cell number decreased to 90.14 ± 17.13, 74.68 ± 10.68, and 66.67 ± 3.29 following 1μM Aβ_1–42_ treatment at day 1, 3, and 7, respectively. Whereas it decreased to 39.75 ± 6.36, 28.00 ± 5.24, and 3.25 ± 1.53 following 10μM Aβ_1–42_ treatment at day 1, 3, and 7, respectively. The morphological changes were also observed in differentiated cultures in the first day of incubation, especially in 10μM Aβ_1–42_. The neurite outgrowth rapidly diminished and could not be determined since day 3 (Figs 2B and 3). We found that 1μM Aβ_1–42_ exhibited lower toxicity on neurite outgrowth retraction. The relative neurite outgrowth decreased to 71.67 ± 8.14, 45.90 ± 8.40, and 23.04 ± 4.34% following 1, 3, and 7 days treatments. These results suggested that the neurite retraction was rapidly observed and could be used as a parameter to monitor neurotoxicity against Aβ_1–42._ Following the results from this experiment, the dose of 1μM Aβ_1–42_ treatment for 1 day was, therefore, used in further study.

### LLLT attenuated Aβ_1-42_-induced neurotoxicity and recovered neurite outgrowth

**Fig 4:**
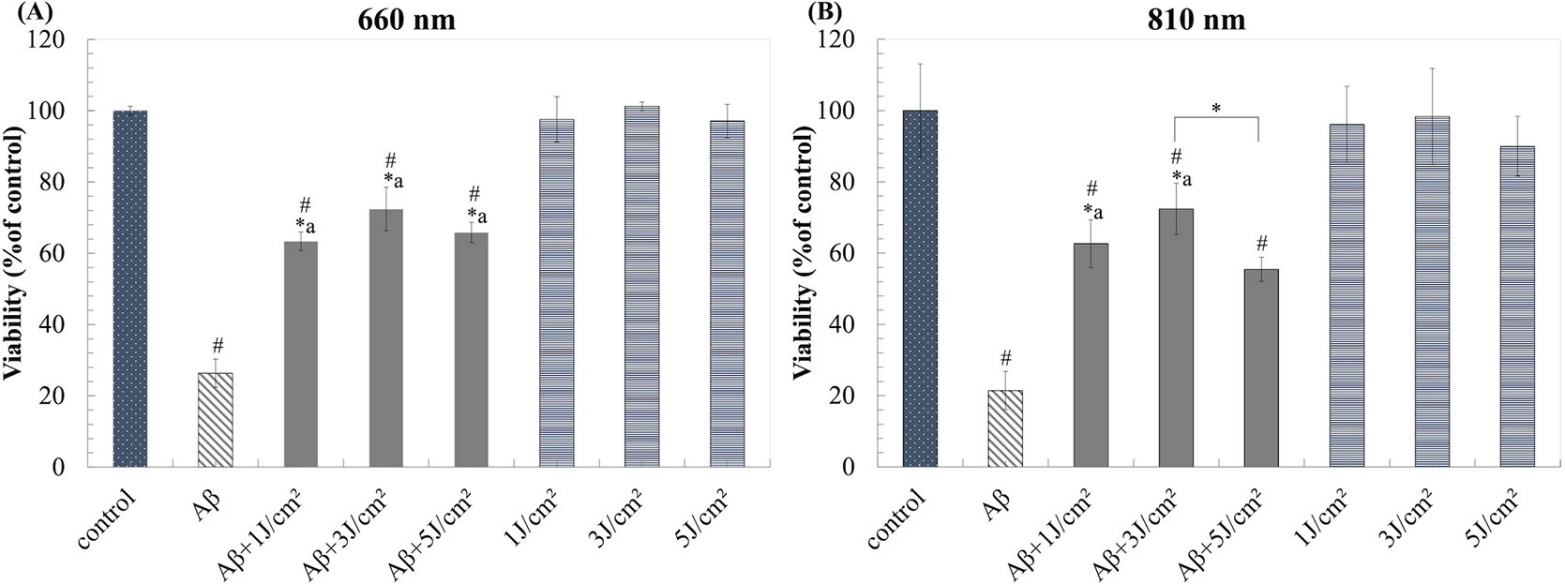
LLLT reduces Aβ_1-42_-induced toxicity in neuronal cells after 7 days of irradiation. (A) and (B) Viability (% of control) after treatment with 660 nm and 810 nm, respectively. The values are means ± SD; n = 3. *P<0.05 for the determined groups. *a P<0.05 vs Aβ. #P<0.05 vs control.

To assess the neuroprotective effects of LLLT, differentiated neurons were induced with1μM Aβ_1–42_ for 1 day then further treated with LLLT for 7 days (once a day). The LEDs planar array of 660 nm and 810 nm, constant power density of 5 mW/cm^2^, and energy density of 1, 3, and 5 J/cm^2^ were used for irradiation. Based on the ability of LLLT to modulate mitochondrial function, we therefore measured cell viability of cell by using trypan blue exclusion assay instead of using MTT assay. The result showed that both of LLLT did not cause noticeable toxic to differentiated neurons, while both LLLT could attenuate Aβ_1-42_-induced neurotoxicity. The neuroprotective effect was observed in a biphasic manner at 3 J/cm^2^ with the viability of 72.43% ± 6.10 and 72.42% ± 7.20 at 660 nm and 810 nm, respectively (Fig 4).

**Fig 5:**
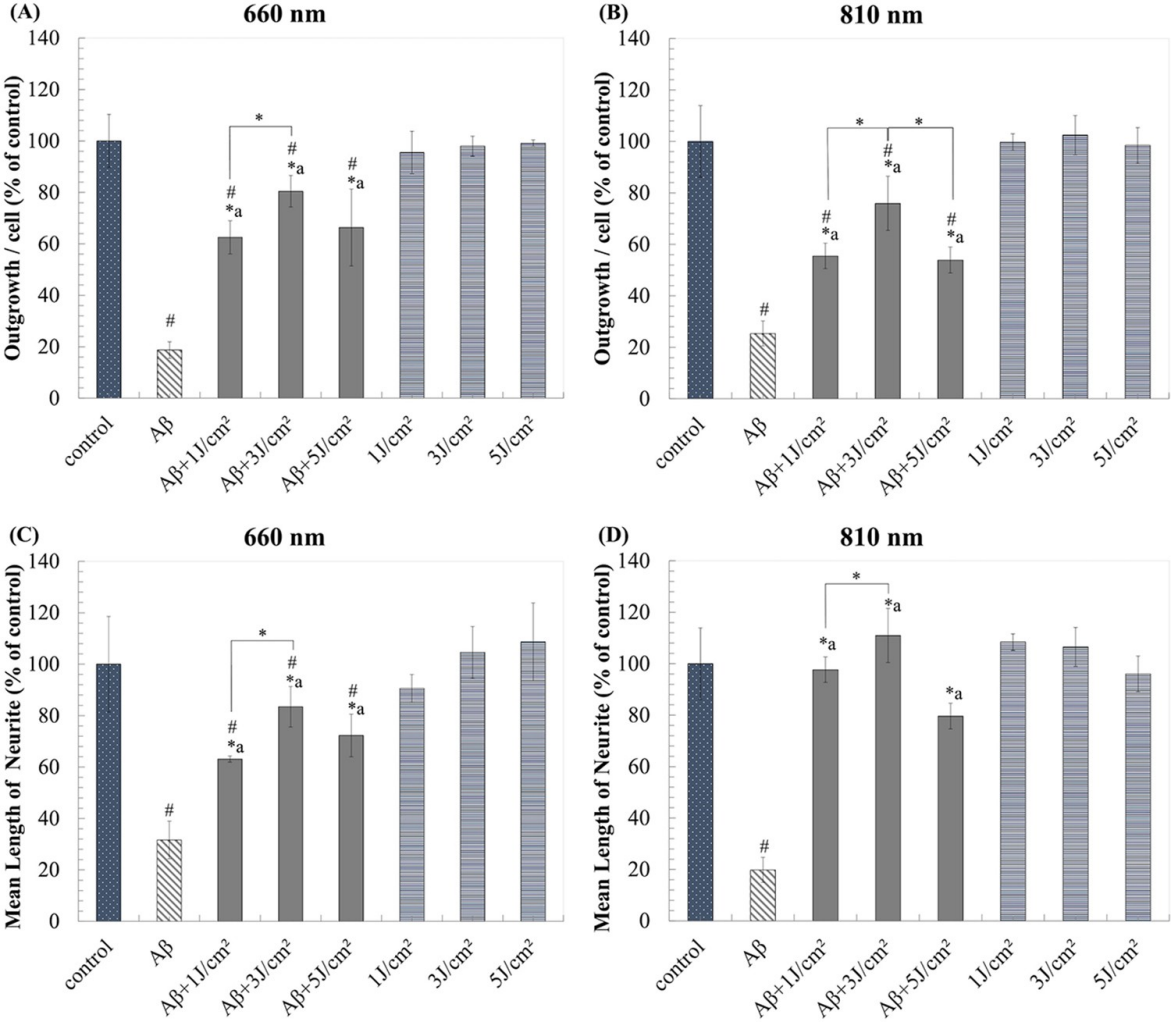
LLLT effects on neurite outgrowth and elongation induced by Aβ_1–42_ toxicity after 7 days of irradiation. (A) and (B) Outgrowth per cell (% of control) after treatment with 660 nm and 810 nm, respectively. (C) and (D) Mean length of neurite (% of control) after treatment with 660 nm and 810 nm, respectively. Data are expressed as mean ± SD; n = 3. *P<0.05 for the determined groups. *a P<0.05 vs Aβ. #P<0.05 vs control.

In addition to the recovery of cell viability, the percentage of neurite outgrowth and length of neurite were monitored. We found that the retraction of neurite outgrowth upon the presence of Aβ_1–42_ was recovered by both LLLT irradiations. The results showed that Aβ_1–42_ reduced neurite outgrowth more than 80% comparing with negative control, while both LLLT could significantly recover the neurite outgrowth number and neurite length (Fig 5). The effects of both LLLT on neurite outgrowth and elongation were in a biphasic manner with a peak at 3 J/cm^2^. As shown in Fig 5A and 5B, 660 nm and 810 nm LLLT at 3 J/cm^2^ could recover neuron outgrowth from Aβ-induced toxicity from about 20% to 80.44% ± 6.14 and 75.91% ± 3.39, respectively. Besides, 3 J/cm^2^ of 660 nm and 810 nm LLLT alone minimally alter neurite length to 104.61% ± 10.05 (P-value = 0.724) and 106.54% ± 7.62 (P-value = 0.514), respectively comparing with negative control (Fig 5C and 5D). We found that LLLT attenuated the neurite outgrowth retraction of differentiated neuron under the absent of Aβ_1–42_. This supported that LLLT is a non-invasive therapeutic method that is not harmful to the cells and can be used to protect neurons from Aβ_1–42_ toxicity.

### LLLT attenuated the mitochondrial depolarization upon Aβ_1–42_ treatment

**Fig 6:**
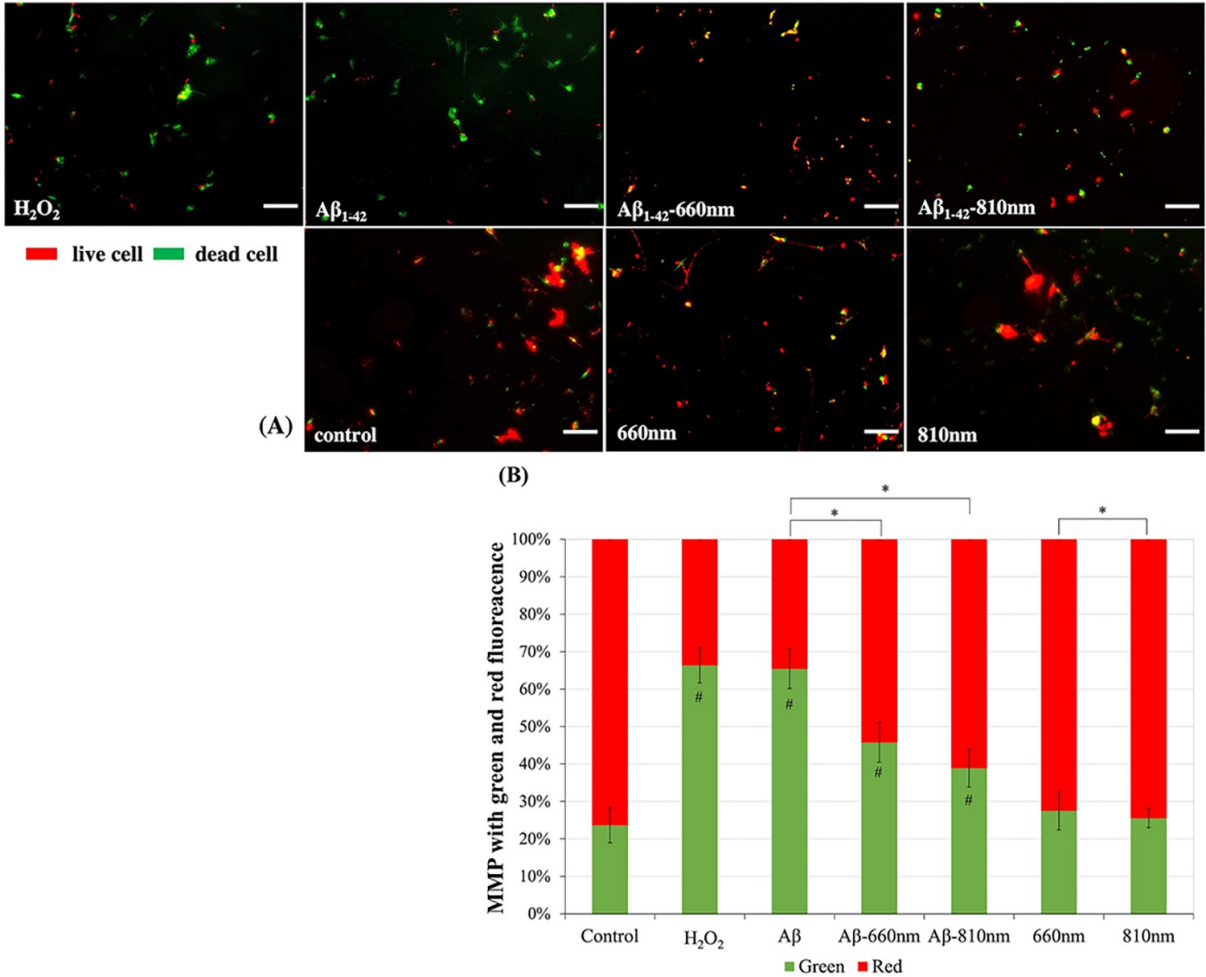
LLLT modulates depolarized mitochondria in neuronal cells induced by Aβ_1–42_ toxicity after 3 days of irradiation. (A) Representative JC-1-stained fluorescent images were obtained with a fluorescence microscope (scale bar represents 100μm). (B) Red and green fluorescence intensity were quantified by using image analysis. Data were obtained on day4 and expressed as mean ± SD; n = 4. *P<0.05 for the determined groups. #P<0.05 vs control.

Based on the mitochondrial modulation capacity of LLLT, in this study we measured the MMP upon Aβ_1–42_ and LLLT exposure. MMP is the change of potential between two layers. As a result of calcium influx, it results in increasing MMP (depolarization). In general, MMP is monitored by JC-1 dye. It indicates by the shift from green fluorescence to red fluorescence. Thus, the depolarization is indicated by the decrease of red/green fluorescence intensity ratio. Previous study revealed that the oxidative stress led to the cytotoxicity by disrupting MMP and stimulate cell apoptosis. We showed that Aβ_1–42_ oligomer could induce cytotoxicity as similar as H_2_O_2_ (known potential oxidant). The relative intensity ratio of red/green fluorescence were reduced to about 34.60 ± 5.07% (P<0.01) and 33.68 ± 5.30% (P<0.01) in Aβ_1–42_ and H_2_O_2_, respectively (Fig 6). The exposure of LLLT in the absent of Aβ_1–42_ did not statistically alter the fluorescence intensity ratio as well as cell viability. Interestingly, the LLLT irradiation following Aβ_1–42_ exposure had ability to attenuate the mitochondrial depolarization about 54.26 ± 7.89% in 660 nm and 61.10 ± 2.50% in 810 nm comparing to Aβ_1–42_ alone. Previous study showed that LLLT had modulated the mitochondria by interacting with photo-acceptors such as cytochromes, water, lipids, S-nitrosylated nitric oxide (NO) and transient receptor potential channels (TRPC) for Ca^2+^. These interaction occurred through various pathways [27]. Thus, the protective effect of LLLT may be due to the alteration of ions across mitochondrial inner membrane which attenuated the Aβ_1–42_ induced-mitochondrial depolarization.

### LLLT irradiation diminished the ROS production upon Aβ_1–42_ exposure

**Fig 7:**
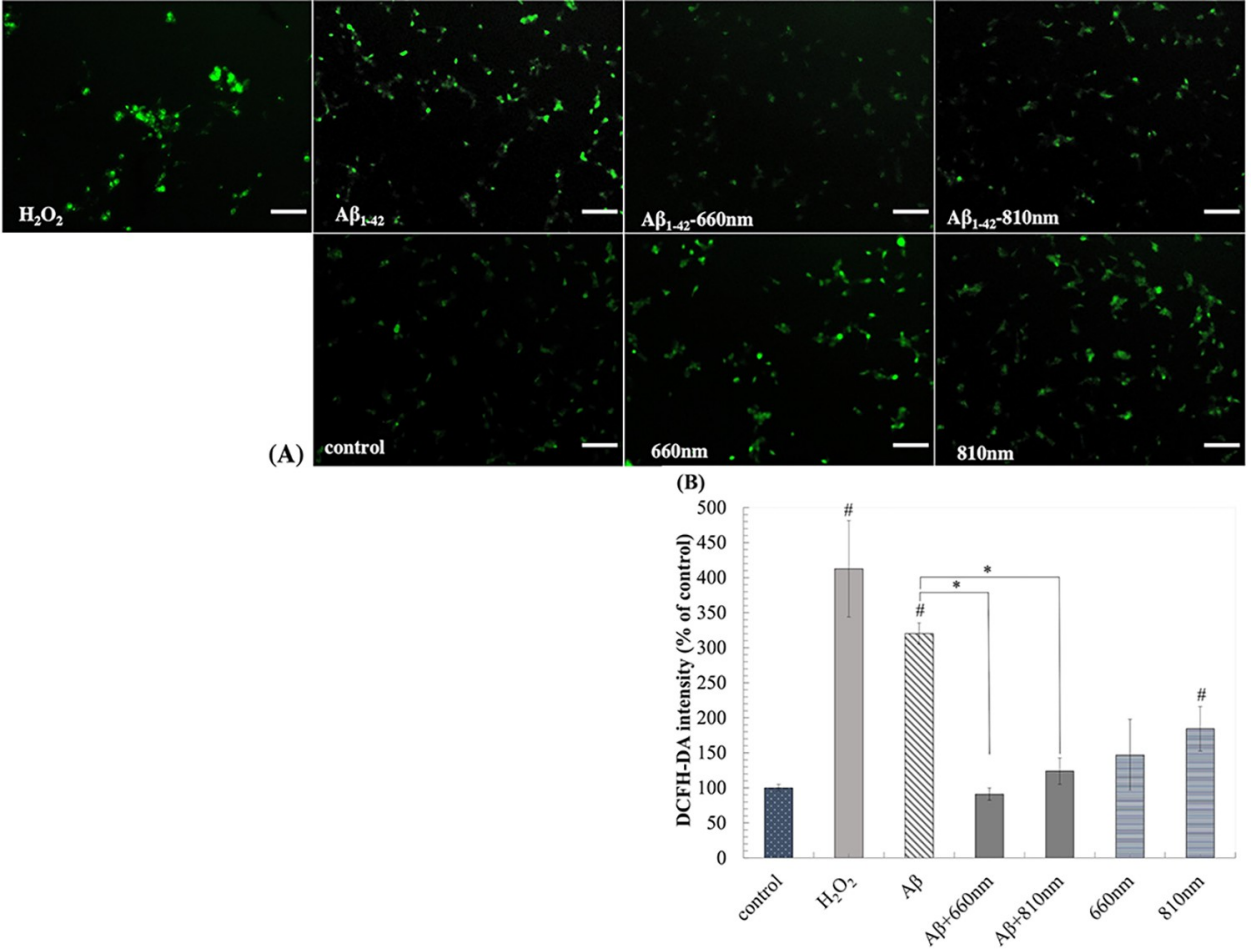
LLLT reduces intracellular ROS induced by Aβ_1-42_-toxicity after 3 days of irradiation. (A) Representative DCFH-DA stained fluorescent images taken with a fluorescence microscope (scale bars represent 100μm). (B) Intensity quantification by image analysis (% of control). Data were obtained on day4 and expressed as mean ± SD; n = 3. *P<0.05 for the determined groups. #P<0.05 vs control.

Previous study revealed that Aβ_1-42_-induced neural toxicity through the elevation of oxidative stress. We found that ROS, determined by DCFH-DA staining, rise to 320.30 ± 15.02% following Aβ_1–42_ exposure. The elevation of ROS upon Aβ_1–42_ was similar to H_2_O_2_, and both conditions rise the intracellular ROS to 3.20 ± 0.15 folds (P<0.01) and 4.12 ± 0.68 folds (P<0.01), respectively comparing with negative control (Fig 7B). We showed that the irradiation of LLLT both wavelengths could attenuate the elevation of intracellular ROS upon Aβ_1–42_ treatment. The LLLT of 660 nm and 810 nm showed therapeutic effects by significantly reduced ROS levels to 0.91 and 1.23 folds, respectively. Interestingly, both LLLT itself could also stimulate intracellular ROS. The LLLT of 660 nm increased ROS production to 1.47 folds, while 810 nm increased ROS to 1.84 folds (P-value = 0.010). The increase of ROS in LLLT alone did not cause cell death. Thus, we believed that ROS may be one of the players that act as a secondary messenger to modulate the cellular response. This level of ROS was lower than ROS from Aβ_1–42_ and H_2_O_2_ treatments. While the LLLT treatment following Aβ_1–42_ did not exert the ROS level, in contrast, in lowered the intracellular ROS and recovered cell death and neurite outgrowth (Figs 4 and 5).

### LLLT lessen the intracellular calcium and protected neuron from Aβ_1-42_-induced toxicity

**Fig 8:**
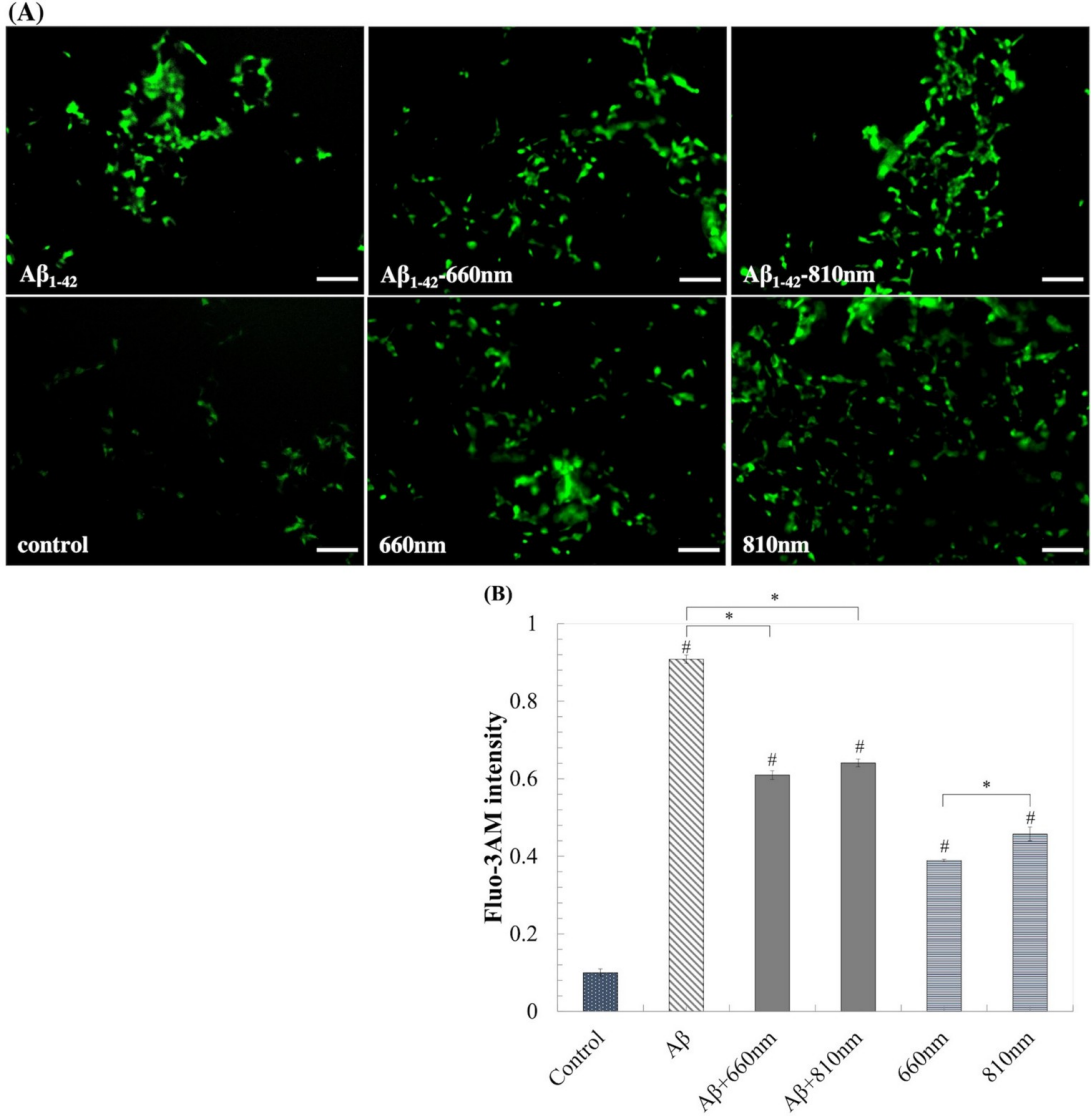
LLLT reduces intracellular Ca^2+^ influx induced by Aβ_1–42_ toxicity after 3 days of irradiation. (A) Representative Fluo-3AM-stained fluorescent images taken with a fluorescence microscope (scale bars represent 100μm). (B) Fluo-3 AM intensity quantification by TECAN. Data were obtained at day4 and were expressed as mean ± SEM; n = 3. *P<0.05 for the determined groups. #P<0.05 vs control.

In this study intracellular calcium (Ca^2+^_i_) was monitored by Fluo-3 fluorescence intensity. Due to the transient response of Ca^2+^_i_ upon LLLT irradiation, we monitored the Ca^2+^_i_ at 10 minutes after exposure to the light. We showed that LLLT alone could induced Ca^2+^_i_ to 0.389 ± 0.003 and 0.458 ± 0.178 of LLLT 660 and 810 nm, respectively. While the presence of Aβ_1–42_ for 1 day raised Ca^2+^_i_ to about 9 folds. We found that the exposure of LLLT once a day for four days could retain Ca^2+^_i_ to about 6 folds in both LLLT wavelengths, which were significantly lower than Aβ_1–42_ exposure alone (Fig 8). We found that LLLT-induced Ca^2+^_i_ elevation effect was transient. The concentration of Ca^2+^_i_ was diminished to non-lethal level within one hour, whereas induction of Ca^2+^_i_ following Aβ_1–42_ exposure were prolonged. We believed that calcium ion may be one of the players that stimulated cellular protection process. LLLT alone did not harmful to the cell, while the exposure of LLLT following the cellular damage from Aβ_1–42_ exposure could attenuate neurotoxicity.

## Discussion

Amyloid plaques have been used as one of the histopathological hallmarks of AD (post-mortem) for many decades. In addition, PET amyloid-β imaging has also been used to diagnose AD patients together with memory assessment while they are still alive. Amyloid-beta (Aβ), especially the soluble oligomeric forms of the Aβ_1–42_ peptide, plays critical roles in AD pathogenesis. It exhibits greater toxicity in neuronal cells than their monomer or fibril forms [28, 29]. The accumulation of Aβ_1–42_ oligomer in the brain induced the oxidative stress, mitochondrial dysfunction, and chronic inflammation. This leads to neural network dysfunction and neurodegenerative diseases [30–33]. Thus, oligomeric Aβ_1–42_ had been used to generate AD *in vitro* model. In this study, we induced neuroblastoma cell with RA and BDNF toward differentiated neuron stage [10]. The differentiation model showed neuron-like cell morphology, which suited for studying neurite outgrowth and neural toxicity for Aβ_1-42_-induced AD model. Following the differentiation, neurons were exposed with oligomeric Aβ_1–42_ for 24 hours prior to LLLT irradiation. Cells were treated with LLLT once a day for 7 days in the presence of Aβ_1–42_. We found that LLLT exhibited the therapeutic effects in Aβ_1-42_-induced neural toxicity.

Neurite outgrowth was used as an early marker to investigate the morphological changes and cell death. This is an important process in neuronal development, particularly the *in vitro* neural networks formation and *in vivo* nerves regeneration. We found that Aβ_1–42_ had neurotoxicity in a concentration and time-dependent manner. Following the treatment of oligomeric Aβ_1–42_, intracellular ROS and Ca^2+^ across while mitochondrial were depolarized. Along with the molecular responses, we observed the axon destruction and cell death in our *in vitro* model. In addition, these observations also observed in AD patients and animal models in several previous studies [34–38].

The molecular mechanisms underlying neuroprotective effects of LLLT might associated with the modulation of intracellular ROS and Ca^2+^. Previous study revealed that, Aβ could accelerate the mitochondria swelling by Ca^2+^ overload through the opening of the mitochondrial permeability transition pore (mPTP) opening [39]. The opened mPTP were induced by Ca^2+^ signaling and ROS which subsequently led to mitochondrial depolarization [40–43]. Previous study in the hippocampal neuron and astrocytes revealed that the arise of oxidative stress and mitochondrial dysfunction related to the activation of NADH oxidase [44]. In this study, oligomeric Aβ_1–42_ could induce intracellular ROS about 3 folds and changed mitochondrial health (determined by JC-1 staining) about 35%. This ROS overproduction and collapse of mitochondrial membrane potential were similarly to the treatment of H_2_O_2_, a membrane-permeable reactive oxygen species.

Mitochondria is the critical energy resource of the living cells. It is known to be the primary photoreceptor of LLLT. Several previous studies showed that red and NIR LLLT activated ATP production, reduced oxidative stress, and enhanced the sprouting of neurite outgrowth [45–47]. The photo-biomodulation may be described by the transfer of electron from LLLT that modulate redox cycling and secondary messenger accumulation. LLLT has been shown to enhance mitochondrial respiration and activate the redox-sensitive NF-kB signaling via the generation of ROS [48]. In addition, NIR light treatment effectively reduced synaptic vulnerability to Aβ oligomers by moderating the binding of Aβ on synaptosomes and increasing synaptic mitochondria *in vivo* [49].

LEDs has been reported as a good light source for emitting red and NIR wavelengths. Due to the interference phenomenon and the excellent tissue scattering characteristics of light at these wavebands, LED had high efficacy of cell penetration [50]. However, the different wavebands could display different bio-stimulation mechanisms. LLLT at 980 nm targeted temperature-gated calcium ion channels, while 810 nm targeted mitochondrial cytochrome c oxidase inhuman adipose-derived stem cells [51]. Neurite retraction in N2a cell following blue-light (473 nm) exposure could be recovered by spotting the red-light (650 nm) on the soma near the junction of retraction site. Furthermore, green-light (550 nm) also had protective effect but lower than red-light. Previous study showed that the stimulation of neurite extension by red-light was associated with the Ca^2+^ influx, actin propagation and myosin II inhibition [52]. In addition, NIR light (808 nm) could promote the neuronal growth of trigeminal ganglion neurons through the activation of mitochondria [53, 54]. Moreover, LLLT with 660 nm and 810 nm at the dose of 3 J/cm^2^ had been reported to increase ATP, raised MMP, and stimulated proliferation rate of hASCs in a relatively similar fashion [55]. Although red and NIR light have been reported to activate cell function, the effects of these wavebands on the cellular level of neurons are still unclear, and the optimal conditions for fluences and irradiance of light exposure were varied depending on the cell type and pathological condition. Our findings showed that a density of 3 J/cm^2^ could recover viability, neurite outgrowth and neurite elongation upon the presence of Aβ_1–42_. However, the higher density of 5 J/cm^2^ and the lower density of 1 J/cm^2^ showed lower neuroprotective effects. These effects were appeared as biphasic manner, which were previously observed in other study [56]. LLLT at 810 nm with fluences of 3 J/cm^2^ showed a biphasic-dose response, which can induce a significant increase in intracellular calcium, ATP, and MMP, as well as stimulate modest levels of ROS that activate signaling pathways. However, these effects decline at higher fluences of 30 J/cm^2^, and can result in a significant increase in ROS levels that induce impaired mitochondrial function and initiate neural death. Previous research has also shown that LLLT at 810 nm with 3 J/cm^2^ can elevate MMP and ROS in normal neurons. Conversely, in oxidatively-stressed cells, LLLT can increase MMP and reduce ROS, thereby protecting against neural death [57]. In this study, both wavelengths; 660 nm and 810 nm were not only restored of neurite outgrowth but also attenuated the Aβ-induced toxicity at all light doses. They exhibited unnoticeable toxicity to the differentiated neurons (Figs 4 and 5). Based on the findings, we suggested that if the irradiation time was too long, the heat was accumulate and led to cellular damage. On the other hand, if the irradiation time was too short, the treatment would be ineffective due to an insufficient photon density to reach the target chromophore. Thus, the therapeutic use of LLLTs should be restricted their energy density and irradiation time.

**Fig 9:**
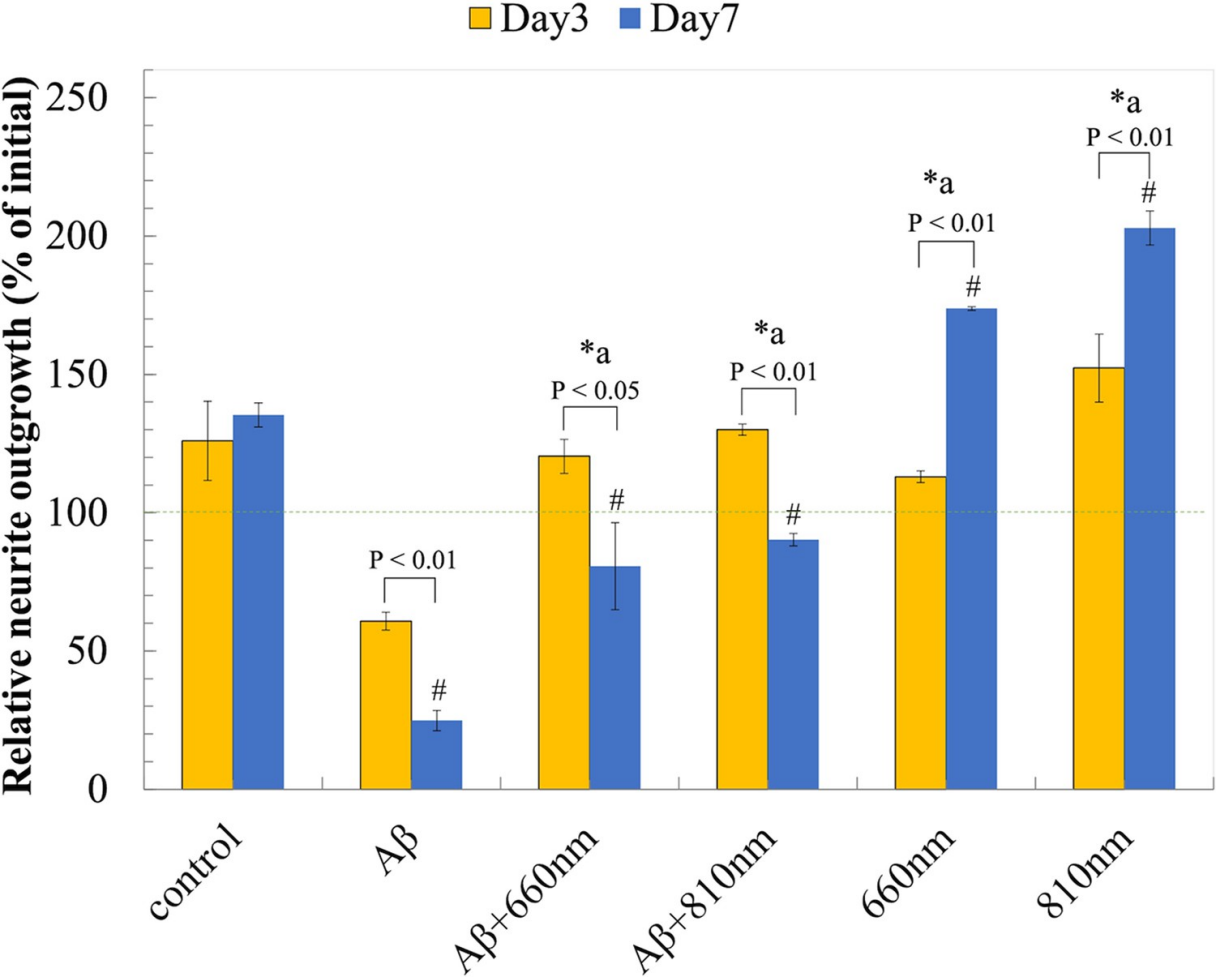
LLLT improves neurite outgrowth. Relative neurite outgrowth (% of control) after LLLT treatment for 3 days and 7 days. Data were expressed as mean ± SD; n = 3. *a P<0.05 vs Aβ. #P<0.05 vs control.

We found that both 810 nm and 660 nm LLLT significantly relieved Aβ_1-42_-induced neuro-toxicity. They improve viability, neurite outgrowth, and neurite elongation upon the irradiation for 3 days, although the LLLT at 810 nm may have greater potential for neuroprotective effects compared to 660 nm (Fig 9). We believed that both red and NIR wavebands could similarly induce mitochondrial photon absorption, intracellular signal transduction and the downstream cellular responses. Based on our observation red and NIR LLLTs was able to recover MMP and improve in cell viability. We showed that the increase of intracellular ROS and Ca^2+^ upon Aβ_1–42_ exposure induced the opening of the mPTP and subsequently led to mitochondrial dysfunction. The high and prolonged increase in cytoplasmic Ca^2+^ levels, up to 500 nM or five-fold over 20 to 30 minutes, was required for neuron death via excitotoxicity [58], while a transient elevation of Ca^2+^ levels from LLLT irradiation may responsible for the bio-stimulatory effects [45]. Previous studies showed that the prolonged opening of the mPTP were associated with a progressive rise of intracellular ROS levels, and elevated Ca^2+^level. These resulted in the degradation of the mitochondria and ultimately program cell death [59–62].

Based on our findings, we suggested that LLLT at 810 nm and 660 nm could stimulate the transient induction of ROS and Ca^2+^, which were present under the sub-lethal amount or lower than the level that causes neuron excitotoxicity. The increase of intracellular ROS and Ca^2+^ levels upon exposure to Aβ_1–42_ was associated with a reduction in MMP, rendering neurons vulnerable to toxicity, physiological changes, and cell death. Conversely, LLLT has the potential to produce the signaling of ROS and Ca^2+^, promoting cellular function and mitochondrial activity, reflected by the increase MMP levels, thus providing cell survival and tolerance to Aβ_1-42_-toxicity.

## Conclusions

**Fig 10:**
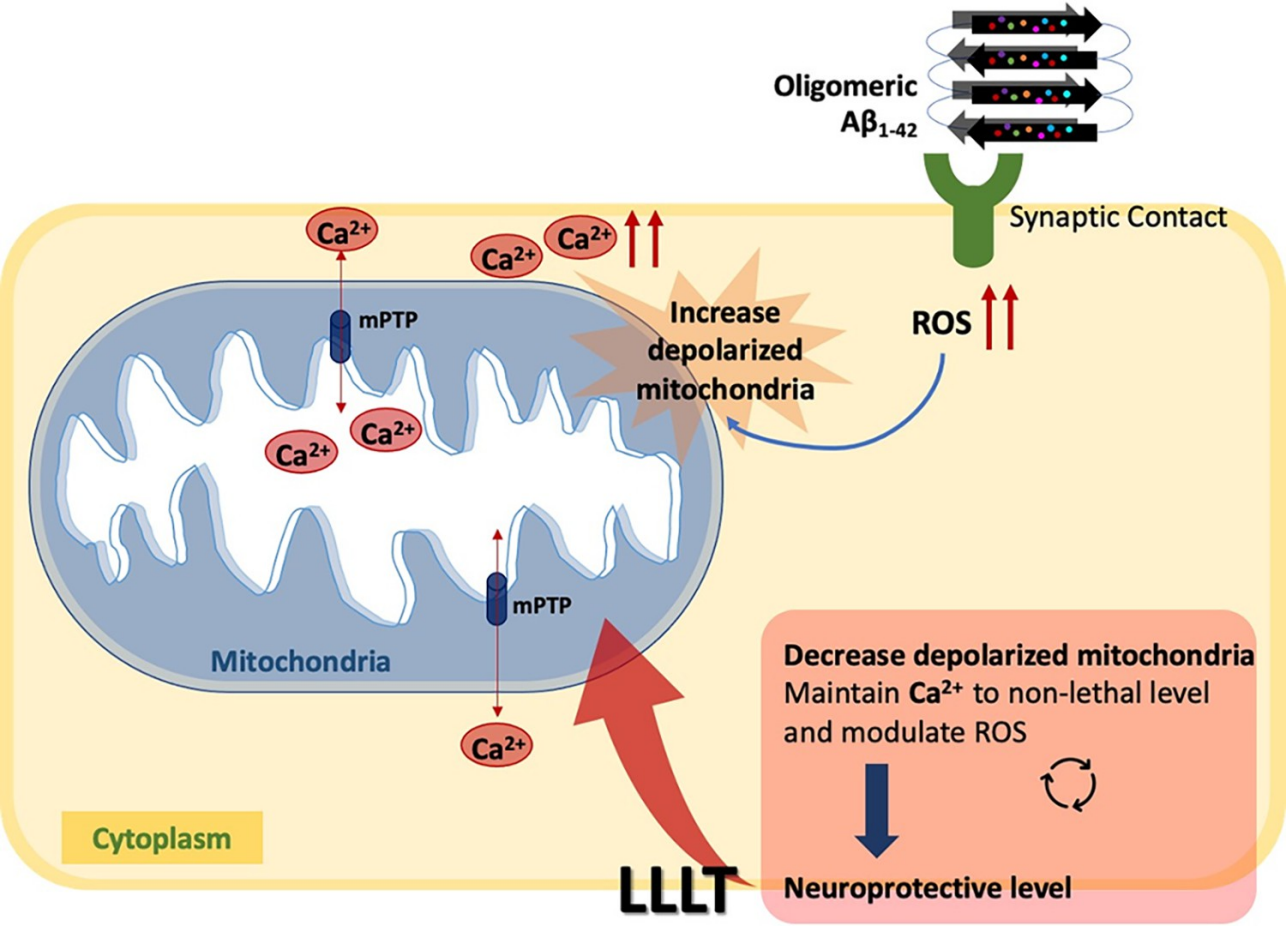
Schematic diagram of the possible mechanism underlying the therapeutic effects of LLLT on Aβ_1-42_-induced neuronal death.

The effects of low intensity 660 nm and 810 nm light on Aβ_1–42_ toxicity in the differentiated neurons have been demonstrated in this study. In this study, LLLT at 660 nm and 810 nm showed a biphasic dose response at 3 J/cm^2^ that could protect Aβ_1–42_ toxicity by recovering neurite outgrowth and cell viability. LLLT could generate the signaling of ROS and Ca^2+^ which in turn promoted downstream cell signaling pathway and provide the beneficial effects for mitochondrial activity (Fig 10). However, the molecular responses upon LLLT have not been determined in this study and should be investigated further in the future study.

## Supporting information

S1 Fig Position of LED on a LEDs array plane simulated with MATLAB for 660-nm and 810-nm LED. (TIF)

S2 Fig Wavelength measurement obtained with a fiber optic spectroradiometer for SMBB660D-1100 and SMBB810N-1100 high power LEDs. The spectral irradiance (μW/cm^2^/nm) at specified wavelengths was then measured with a NIST nm source.
(TIF)

S3 Fig Example of the simulated LED patterns, surface plots of the position of LEDs and the top view of local energy density on the irradiated planes from 660-nm and 810-nm LED arrays when an average energy density at 3 J/cm^2^ is required. (TIFF)

## References

[1] Deshpande A, Mina E, Glabe C, Busciglio J. Different Conformations of Amyloid β Induce Neurotoxicity by Distinct Mechanisms in Human Cortical Neurons. The Journal of Neuroscience. 2006;26(22):6011. 16738244 10.1523/JNEUROSCI.1189-06.2006 PMC6675207

[2] Kayed R, Head E, Thompson JL, McIntire TM, Milton SC, Cotman CW, et al. Common structure of soluble amyloid oligomers implies common mechanism of pathogenesis. Science. 2003;300(5618):486–9. doi: 10.1126/science.1079469 12702875

[3] Morkuniene R, Cizas P, Jankeviciute S, Petrolis R, Arandarcikaite O, Krisciukaitis A, et al. Small Aβ1–42 oligomer‐induced membrane depolarization of neuronal and microglial cells: Role of N‐methyl‐D‐aspartate receptors. Journal of neuroscience research. 2015;93(3):475–86. 25400096 10.1002/jnr.23510

[4] Rao VK, Carlson EA, Yan SS. Mitochondrial permeability transition pore is a potential drug target for neurodegeneration. Biochim Biophys Acta. 2014;1842(8):1267–72. doi: 10.1016/j.bbadis.2013.09.003 24055979 PMC3991756

[5] Demuro A, Parker I, Stutzmann GE. Calcium signaling and amyloid toxicity in Alzheimer disease. J Biol Chem. 2010;285(17):12463–8. doi: 10.1074/jbc.R109.080895 20212036 PMC2857063

[6] Calvo-Rodriguez M, Hernando-Perez E, Nuñez L, Villalobos C. Amyloid β oligomers increase ER-mitochondria Ca2+ cross talk in young hippocampal neurons and exacerbate aging-induced intracellular Ca2+ remodeling. Frontiers in cellular neuroscience. 2019;13:22. 30800057 10.3389/fncel.2019.00022 PMC6376150

[7] Hayashi T, Rizzuto R, Hajnoczky G, Su T-P. MAM: more than just a housekeeper. Trends in cell biology. 2009;19(2):81–8. doi: 10.1016/j.tcb.2008.12.002 19144519 PMC2750097

[8] Mark RJ, Hensley K, Butterfield DA, Mattson MP. Amyloid beta-peptide impairs ion-motive ATPase activities: evidence for a role in loss of neuronal Ca2+ homeostasis and cell death. Journal of Neuroscience. 1995;15(9):6239–49. doi: 10.1523/JNEUROSCI.15-09-06239.1995 7666206 PMC6577674

[9] Krishtal J, Bragina O, Metsla K, Palumaa P, Tõugu V. In situ fibrillizing amyloid-beta 1–42 induces neurite degeneration and apoptosis of differentiated SH-SY5Y cells. PloS one. 2017;12(10):e0186636. doi: 10.1371/journal.pone.0186636 29065138 PMC5655426

[10] Forster J, Köglsberger S, Trefois C, Boyd O, Baumuratov A, Buck L, et al. Characterization of differentiated SH-SY5Y as neuronal screening model reveals increased oxidative vulnerability. Journal of biomolecular screening. 2016;21(5):496–509. doi: 10.1177/1087057115625190 26738520 PMC4904349

[11] Tam SY, Tam VC, Ramkumar S, Khaw ML, Law HK, Lee SW. Review on the cellular mechanisms of low-level laser therapy use in oncology. Frontiers in Oncology. 2020:1255. doi: 10.3389/fonc.2020.01255 32793501 PMC7393265

[12] Hong N. Photobiomodulation as a treatment for neurodegenerative disorders: current and future trends. Biomedical Engineering Letters. 2019;9(3):359–66. doi: 10.1007/s13534-019-00115-x 31456895 PMC6694374

[13] Avci P, Gupta A, Sadasivam M, Vecchio D, Pam Z, Pam N, et al., editors. Low-level laser (light) therapy (LLLT) in skin: stimulating, healing, restoring. Seminars in cutaneous medicine and surgery; 2013: NIH Public Access. 24049929 PMC4126803

[14] de Freitas LF, Hamblin MR. Proposed mechanisms of photobiomodulation or low-level light therapy. IEEE Journal of selected topics in quantum electronics. 2016;22(3):348–64. doi: 10.1109/JSTQE.2016.2561201 28070154 PMC5215870

[15] Hamblin MR. Mechanisms and Mitochondrial Redox Signaling in Photobiomodulation. Photochem Photobiol. 2018;94(2):199–212. doi: 10.1111/php.12864 29164625 PMC5844808

[16] Levchenko SM, Kuzmin AN, Ohulchanskyy TY, Pliss A, Qu J, Prasad PN. Near-Infrared Irradiation Affects Lipid Metabolism in Neuronal Cells, Inducing Lipid Droplets Formation. ACS Chem Neurosci. 2019;10(3):1517–23. doi: 10.1021/acschemneuro.8b00508 30499655

[17] Golovynska I, Golovynskyi S, Stepanov YV, Stepanova LI, Qu J, Ohulchanskyy TY. Red and near-infrared light evokes Ca2+ influx, endoplasmic reticulum release and membrane depolarization in neurons and cancer cells. Journal of Photochemistry and Photobiology B: Biology. 2021;214:112088. 33278762 10.1016/j.jphotobiol.2020.112088

[18] Wong-Riley MT, Liang HL, Eells JT, Chance B, Henry MM, Buchmann E, et al. Photobiomodulation directly benefits primary neurons functionally inactivated by toxins: role of cytochrome c oxidase. Journal of Biological Chemistry. 2005;280(6):4761–71. doi: 10.1074/jbc.M409650200 15557336

[19] Huang YY, Nagata K, Tedford CE, Hamblin MR. Low‐level laser therapy (810 nm) protects primary cortical neurons against excitotoxicity in vitro. Journal of biophotonics. 2014;7(8):656–64. doi: 10.1002/jbio.201300125 24127337 PMC4057365

[20] Amaroli A, Ferrando S, Benedicenti S. Photobiomodulation affects key cellular pathways of all life‐forms: considerations on old and new laser light targets and the calcium issue. Photochemistry and photobiology. 2019;95(1):455–9. doi: 10.1111/php.13032 30281800

[21] Ureshino RP, Erustes AG, Bassani TB, Wachilewski P, Guarache GC, Nascimento AC, et al. The interplay between Ca2+ signaling pathways and neurodegeneration. International journal of molecular sciences. 2019;20(23):6004. doi: 10.3390/ijms20236004 31795242 PMC6928941

[22] Stine WB, Jungbauer L, Yu C, LaDu MJ. Preparing synthetic Aβ in different aggregation states. Alzheimer’s Disease and Frontotemporal Dementia: Methods and Protocols. 2011:13–32. 10.1007/978-1-60761-744-0_2 PMC3752843 20967580

[23] Kim H, Xue X. Detection of Total Reactive Oxygen Species in Adherent Cells by 2’,7’-Dichlorodihydrofluorescein Diacetate Staining. J Vis Exp. 2020(160). doi: 10.3791/60682 32658187 PMC7712457

[24] Sivandzade F, Bhalerao A, Cucullo L. Analysis of the Mitochondrial Membrane Potential Using the Cationic JC-1 Dye as a Sensitive Fluorescent Probe. Bio Protoc. 2019;9(1). 10.21769/BioProtoc.3128 PMC6343665 30687773

[25] Boulan B, Beghin A, Ravanello C, Deloulme J-C, Gory-Fauré S, Andrieux A, et al. AutoNeuriteJ: An ImageJ plugin for measurement and classification of neuritic extensions. PLoS One. 2020;15(7):e0234529. doi: 10.1371/journal.pone.0234529 32673338 PMC7365462

[26] Pemberton K, Mersman B, Xu F. Using ImageJ to assess neurite outgrowth in mammalian cell cultures: research data quantification exercises in undergraduate neuroscience lab. Journal of Undergraduate Neuroscience Education. 2018;16(2):A186. doi: 10.1159/000207490 30057501 PMC6057772

[27] Ravera S, Colombo E, Pasquale C, Benedicenti S, Solimei L, Signore A, et al. Mitochondrial bioenergetic, photobiomodulation and trigeminal branches nerve damage, what’s the connection? A Review. International Journal of Molecular Sciences. 2021;22(9):4347. doi: 10.3390/ijms22094347 33919443 PMC8122620

[28] Lee JY, Park Y, Pun S, Lee SS, Lo JF, Lee LP. Real-time investigation of cytochrome c release profiles in living neuronal cells undergoing amyloid beta oligomer-induced apoptosis. Nanoscale. 2015;7(23):10340–3. doi: 10.1039/c5nr02390d 26009283

[29] Gandhi S, Refolo LM, Sambamurti K. Amyloid precursor protein compartmentalization restricts β-amyloid production: therapeutic targets based on BACE compartmentalization. Journal of Molecular Neuroscience. 2004;24:137–43. 15314262 10.1385/JMN:24:1:137

[30] Schilling T, Eder C. Amyloid‐β‐induced reactive oxygen species production and priming are differentially regulated by ion channels in microglia. Journal of cellular physiology. 2011;226(12):3295–302. 21321937 10.1002/jcp.22675

[31] Manczak M, Reddy PH. Abnormal interaction of oligomeric amyloid-β with phosphorylated tau: implications to synaptic dysfunction and neuronal damage. Journal of Alzheimer’s Disease. 2013;36(2):285–95. 10.3233/JAD-130275 PMC3943249 23594602

[32] Federico A, Cardaioli E, Da Pozzo P, Formichi P, Gallus GN, Radi E. Mitochondria, oxidative stress and neurodegeneration. Journal of the neurological sciences. 2012;322(1–2):254–62. doi: 10.1016/j.jns.2012.05.030 22669122

[33] Song M-S, Saavedra L, de Chaves EIP. Apoptosis is secondary to non-apoptotic axonal degeneration in neurons exposed to Aβ in distal axons. Neurobiology of aging. 2006;27(9):1224–38. 16122841 10.1016/j.neurobiolaging.2005.06.007

[34] Kook S-Y, Hong HS, Moon M, Ha CM, Chang S, Mook-Jung I. Aβ&lt;sub&gt;1–42&lt;/sub&gt;-RAGE Interaction Disrupts Tight Junctions of the Blood–Brain Barrier Via Ca&lt;sup&gt;2+&lt;/sup&gt;-Calcineurin Signaling. The Journal of Neuroscience. 2012;32(26):8845. 22745485 10.1523/JNEUROSCI.6102-11.2012 PMC6622350

[35] Oláh J, Vincze O, Virók D, Simon D, Bozsó Z, Tőkési N, et al. Interactions of pathological hallmark proteins: Tubulin polymerization promoting protein/p25, β-amyloid, and α-synuclein. Journal of Biological Chemistry. 2011;286(39):34088–100. 21832049 10.1074/jbc.M111.243907 PMC3190826

[36] Tobore TO. On the central role of mitochondria dysfunction and oxidative stress in Alzheimer’s disease. Neurological Sciences. 2019;40:1527–40. doi: 10.1007/s10072-019-03863-x 30982132

[37] Popugaeva E, Pchitskaya E, Bezprozvanny I. Dysregulation of neuronal calcium homeostasis in Alzheimer’s disease–A therapeutic opportunity? Biochemical and Biophysical Research Communications. 2017;483(4):998–1004. doi: 10.1016/j.bbrc.2016.09.053 27641664 PMC5303663

[38] Angelova PR, Abramov AY. Alpha-synuclein and beta-amyloid–different targets, same players: calcium, free radicals and mitochondria in the mechanism of neurodegeneration. Biochemical and biophysical research communications. 2017;483(4):1110–5. doi: 10.1016/j.bbrc.2016.07.103 27470584

[39] Jang S, Chapa-Dubocq XR, Parodi-Rullán RM, Fossati S, Javadov S. Beta-amyloid instigates dysfunction of mitochondria in cardiac cells. Cells. 2022;11(3):373. doi: 10.3390/cells11030373 35159183 PMC8834545

[40] Abeti R, Abramov AY. Mitochondrial Ca2+ in neurodegenerative disorders. Pharmacological Research. 2015;99:377–81. doi: 10.1016/j.phrs.2015.05.007 26013908

[41] Tong Y, Bai L, Gong R, Chuan J, Duan X, Zhu Y. Shikonin protects PC12 cells against β-amyloid peptide-induced cell injury through antioxidant and antiapoptotic activities. Scientific reports. 2018;8(1):26. 29311595 10.1038/s41598-017-18058-7 PMC5758797

[42] Bu Islam, Jabir NR, Tabrez S. The role of mitochondrial defects and oxidative stress in Alzheimer’s disease. Journal of drug targeting. 2019;27(9):932–42. doi: 10.1080/1061186X.2019.1584808 30775938

[43] Xu J, Zhou L, Weng Q, Xiao L, Li Q. Curcumin analogues attenuate Aβ25-35-induced oxidative stress in PC12 cells via Keap1/Nrf2/HO-1 signaling pathways. Chemico-biological interactions. 2019;305:171–9. 30946834 10.1016/j.cbi.2019.01.010

[44] Abramov AY, Canevari L, Duchen MR. β-amyloid peptides induce mitochondrial dysfunction and oxidative stress in astrocytes and death of neurons through activation of NADPH oxidase. Journal of Neuroscience. 2004;24(2):565–75. 14724257 10.1523/JNEUROSCI.4042-03.2004 PMC6729998

[45] Lubart R, Lavi R, Friedmann H, Rochkind S. Photochemistry and photobiology of light absorption by living cells. Photomedicine and Laser Therapy. 2006;24(2):179–85. doi: 10.1089/pho.2006.24.179 16706696

[46] Heo J-C, Park J-A, Kim D-K, Lee J-H. Photobiomodulation (660 nm) therapy reduces oxidative stress and induces BDNF expression in the hippocampus. Scientific reports. 2019;9(1):10114. 31300736 10.1038/s41598-019-46490-4 PMC6625994

[47] Mohamad SA, Milward MR, Hadis MA, Kuehne SA, Cooper PR. Photobiomodulation of mineralisation in mesenchymal stem cells. Photochemical & Photobiological Sciences. 2021;20(5):699–714. doi: 10.1007/s43630-021-00047-5 33945145

[48] Chen AC, Arany PR, Huang Y-Y, Tomkinson EM, Sharma SK, Kharkwal GB, et al. Low-level laser therapy activates NF-kB via generation of reactive oxygen species in mouse embryonic fibroblasts. PloS one. 2011;6(7):e22453. doi: 10.1371/journal.pone.0022453 21814580 PMC3141042

[49] Comerota MM, Krishnan B, Taglialatela G. Near infrared light decreases synaptic vulnerability to amyloid beta oligomers. Scientific Reports. 2017;7(1):15012. doi: 10.1038/s41598-017-15357-x 29118388 PMC5678170

[50] Huang Y-Y, Sharma SK, Carroll J, Hamblin MR. Biphasic dose response in low level light therapy–an update. Dose-response. 2011;9(4):dose-response. 11–009. Hamblin. doi: 10.2203/dose-response.11-009.Hamblin 22461763 PMC3315174

[51] Wang Y, Huang Y-Y, Wang Y, Lyu P, Hamblin MR. Photobiomodulation of human adipose-derived stem cells using 810nm and 980nm lasers operates via different mechanisms of action. Biochimica et Biophysica Acta (BBA)—General Subjects. 2017;1861(2):441–9. doi: 10.1016/j.bbagen.2016.10.008 27751953 PMC5195895

[52] Kao YC, Liao YC, Cheng PL, Lee CH. Neurite regrowth stimulation by a red-light spot focused on the neuronal cell soma following blue light-induced retraction. Sci Rep. 2019;9(1):18210. doi: 10.1038/s41598-019-54687-w 31796850 PMC6890775

[53] Cho H, Jeon H-J, Park S, Park C-S, Chung E. Neurite growth of trigeminal ganglion neurons in vitro with near-infrared light irradiation. Journal of Photochemistry and Photobiology B: Biology. 2020;210:111959. doi: 10.1016/j.jphotobiol.2020.111959 32739664

[54] Stepanov YV, Golovynska I, Zhang R, Golovynskyi S, Stepanova LI, Gorbach O, et al. Near-infrared light reduces β-amyloid-stimulated microglial toxicity and enhances survival of neurons: mechanisms of light therapy for Alzheimer’s disease. Alzheimer’s Research & Therapy. 2022;14(1):84. 10.1186/s13195-022-01022-7 PMC9206341 35717405

[55] Wang Y, Huang Y-Y, Wang Y, Lyu P, Hamblin MR. Red (660 nm) or near-infrared (810 nm) photobiomodulation stimulates, while blue (415 nm), green (540 nm) light inhibits proliferation in human adipose-derived stem cells. Scientific Reports. 2017;7(1):7781. 28798481 10.1038/s41598-017-07525-w PMC5552860

[56] Sharma SK, Kharkwal GB, Sajo M, Huang YY, De Taboada L, McCarthy T, et al. Dose response effects of 810 nm laser light on mouse primary cortical neurons. Lasers in surgery and medicine. 2011;43(8):851–9. doi: 10.1002/lsm.21100 21956634 PMC3199299

[57] Huang YY, Nagata K, Tedford CE, McCarthy T, Hamblin MR. Low-level laser therapy (LLLT) reduces oxidative stress in primary cortical neurons in vitro. J Biophotonics. 2013;6(10):829–38. doi: 10.1002/jbio.201200157 23281261 PMC3651776

[58] Mattson MP, Cheng B, Davis D, Bryant K, Lieberburg I, Rydel RE. beta-Amyloid peptides destabilize calcium homeostasis and render human cortical neurons vulnerable to excitotoxicity. The Journal of Neuroscience. 1992;12(2):376. doi: 10.1523/JNEUROSCI.12-02-00376.1992 1346802 PMC6575616

[59] Pchitskaya E, Popugaeva E, Bezprozvanny I. Calcium signaling and molecular mechanisms underlying neurodegenerative diseases. Cell Calcium. 2018;70:87–94. doi: 10.1016/j.ceca.2017.06.008 28728834 PMC5748019

[60] Zorov DB, Juhaszova M, Sollott SJ. Mitochondrial ROS-induced ROS release: An update and review. Biochimica et Biophysica Acta (BBA)—Bioenergetics. 2006;1757(5):509–17. doi: 10.1016/j.bbabio.2006.04.029 16829228

[61] Zhou L, Aon MA, Liu T, O’Rourke B. Dynamic modulation of Ca2+ sparks by mitochondrial oscillations in isolated guinea pig cardiomyocytes under oxidative stress. J Mol Cell Cardiol. 2011;51(5):632–9. doi: 10.1016/j.yjmcc.2011.05.007 21645518 PMC3179563

[62] Zorov DB, Juhaszova M, Sollott SJ. Mitochondrial reactive oxygen species (ROS) and ROS-induced ROS release. Physiol Rev. 2014;94(3):909–50. doi: 10.1152/physrev.00026.2013 24987008 PMC4101632

