# Supplementary figures and images for: Attenuation Aβ1-42-induced neurotoxicity in neuronal cell by 660nm and 810nm LED light irradiation
Source: PLoS One. 2023 Jul 21;18(7):e0283976. doi: 10.1371/journal.pone.0283976 (PMC10361470; doi:10.1371/journal.pone.0283976)

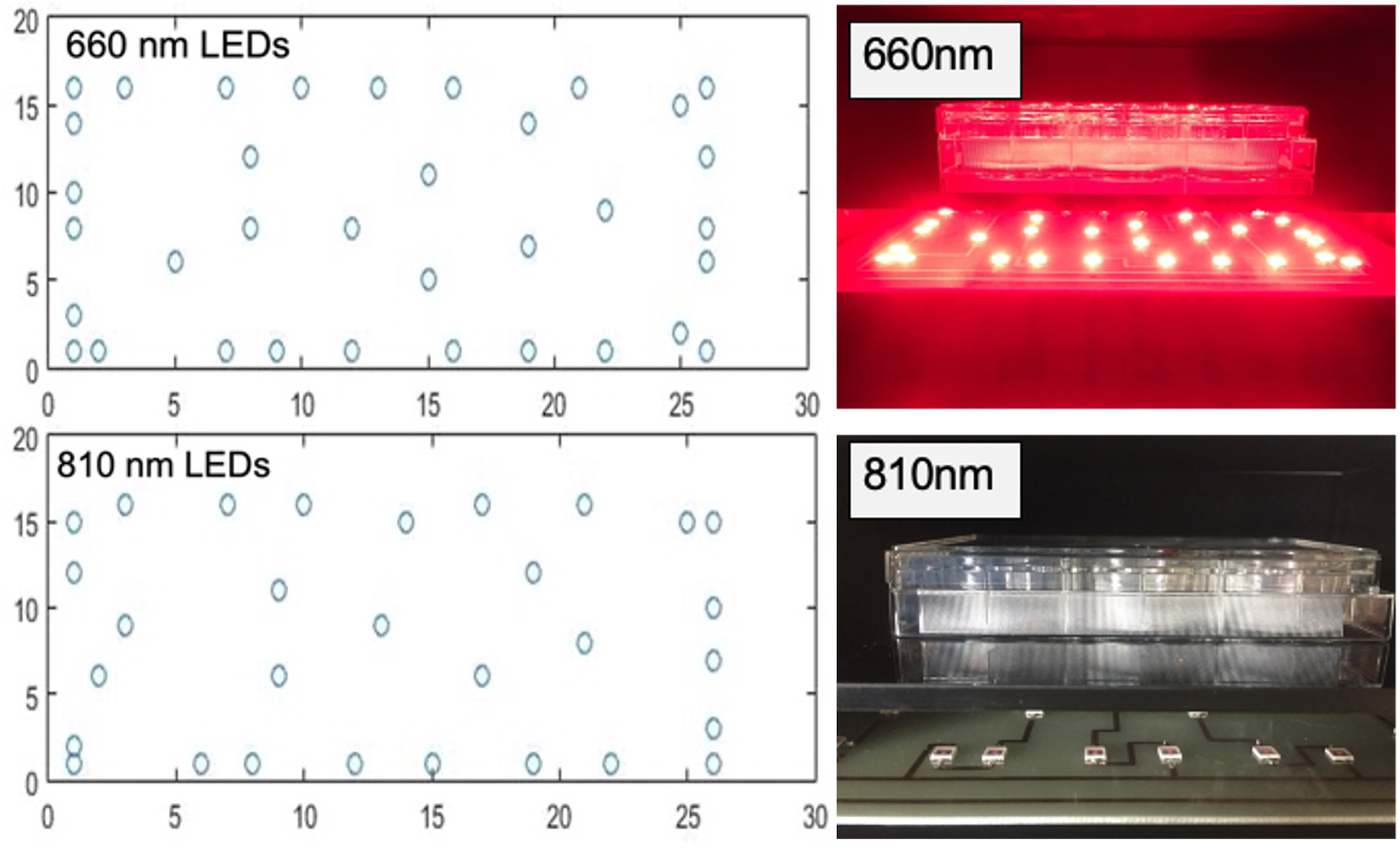

Supplement: S1 Fig — (TIF) [file pone.0283976.s001.tif]

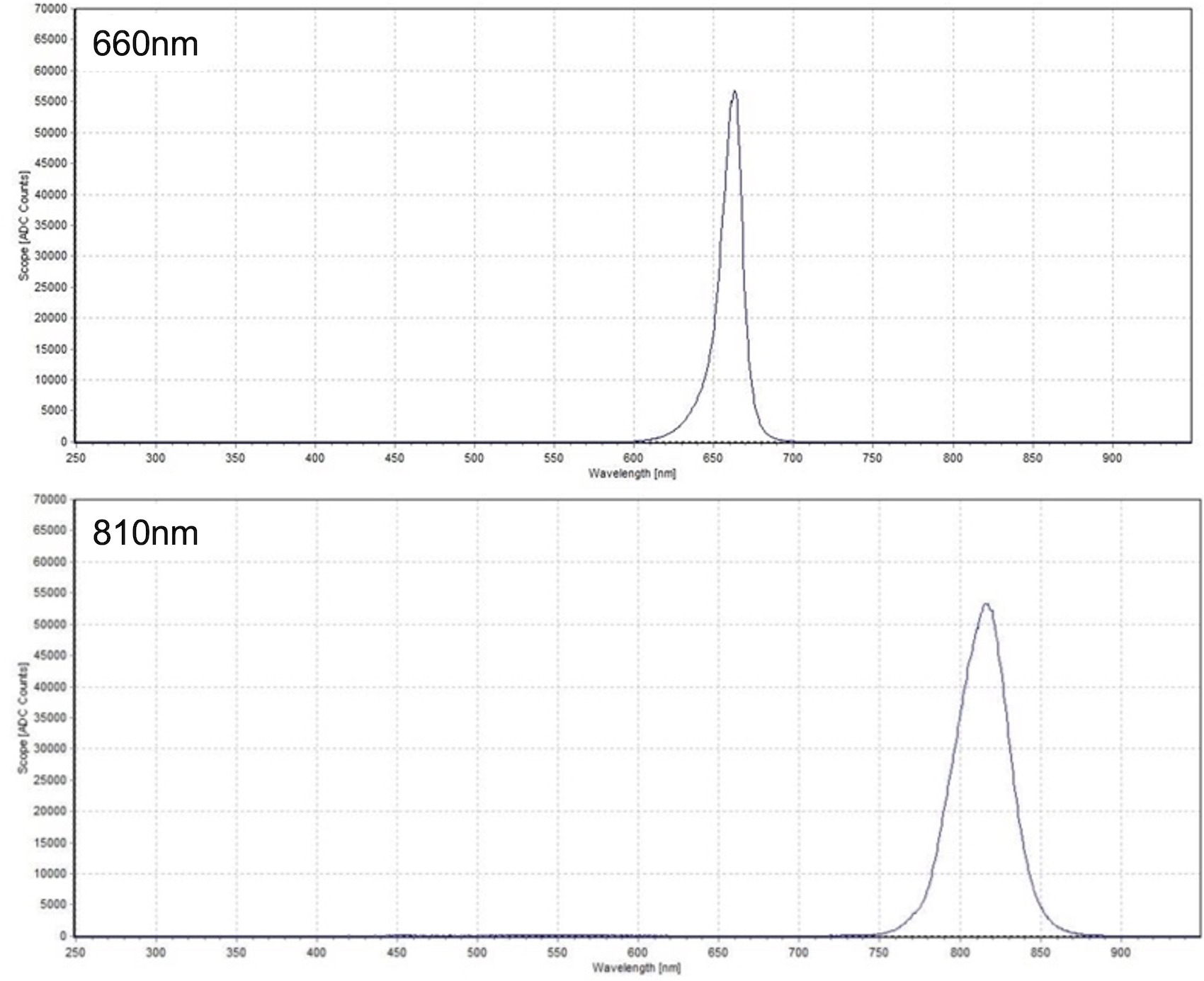

Supplement: S2 Fig — The spectral irradiance (μW/cm2/nm) at specified wavelengths was then measured with a NIST nm source. (TIF) [file pone.0283976.s002.tif]

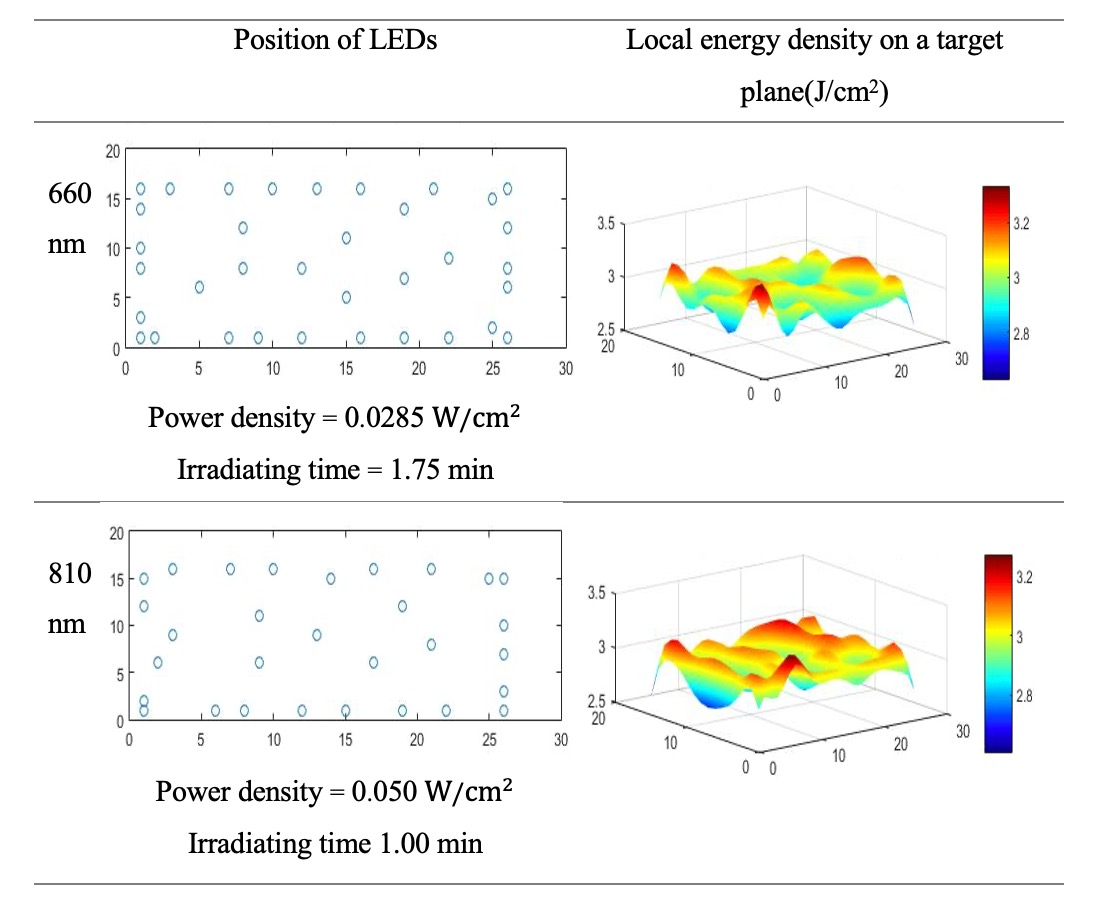

Supplement: S3 Fig — (TIFF) [file pone.0283976.s003.tiff]
